# Comparative analysis of floral transition and floral organ formation in two contrasting species: *Disocactus speciosus* and *D. eichlamii* (Cactaceae)

**DOI:** 10.1007/s00497-023-00494-3

**Published:** 2024-01-09

**Authors:** Cristian Genaro Ramírez-Castro, Alma Piñeyro-Nelson, Estela Sandoval-Zapotitla, Salvador Arias, Isaura Rosas-Reinhold

**Affiliations:** 1 Instituto de Biología, Jardín Botánico, Universidad Nacional Autónoma de México, C.P.04510 Mexico City, Mexico; 2 Departamento de Producción Agrícola y Animal, Universidad Autónoma Metropolitana-Xochimilco, C.P.04510 Mexico City, Mexico; 3 Centro de Ciencias de la Complejidad (C3), Universidad Nacional Autónoma de México, C.P.04960 Mexico City, Mexico; 4 Posgrado en Ciencias Biológicas, Unidad de Posgrado, Edificio D, 1° Piso, Circuito de Posgrados, Ciudad Universitaria, Coyoacán, Mexico City, C.P. 04510 Mexico; 5 Present Address: Center for Genomics and Systems Biology, New York University, 12 Waverly Pl, New York, NY 10003 USA

**Keywords:** Epiphytic cacti, Floral anatomy, Hylocereeae, Pericarpel, Ring meristem

## Abstract

**Key message:**

Contrasting morphologies in *Disocactus* are the result of differential development of the vegetative and floral tissue where intercalary growth is involved, resulting in a complex structure, the floral axis.

**Abstract:**

Species from the Cactaceae bear adaptations related with their growth in environments under hydric stress. These adaptations have translated into the reduction and modification of various structures such as leaves, stems, lateral branches, roots and the structuring of flowers in a so-called *flower-shoot*. While cacti flowers and fruits have a consistent structure with showy hermaphrodite or unisexual flowers that produce a fruit called *cactidium*, the developmental dynamics of vegetative and reproductive tissues comprising the reproductive unit have only been inferred through the analysis of pre-anthetic buds. Here we present a comparative analysis of two developmental series covering the early stages of flower formation and organ differentiation in *Disocactus speciosus* and *Disocactus eichlamii,* which have contrasting floral morphologies. We observe that within the areole, a shoot apical meristem commences to grow upward, producing lateral leaves with a spiral arrangement, rapidly transitioning to a floral meristem. The floral meristem produces tepal primordia and a staminal ring meristem from which numerous or few stamens develop in a centrifugal manner in *D. speciosus* and *D. eichlamii*, respectively. Also, the inferior ovary derives from the floral meristem flattening and an upward growth of the surrounding tissue of the underlying stem, producing the pericarpel. This structure is novel to cacti and lacks a clear anatomical delimitation with the carpel wall. Here, we present a first study that documents the early processes taking place during initial meristem determination related to pericarpel development and early floral organ formation in cacti until the establishment of mature floral organs.

## Introduction

The emergence of the flower during angiosperm evolution represented a hiatus that was the basis for the rapid process of radiation and diversification documented in flowering plants, which are the dominating plant group in the world (Moyroud and Glover 2017). The diversification of floral structures and the evolution of different pollination syndromes have been credited as two of the main forces underlying the success of flowering plants (Moyroud and Glover 2017).

Structural innovations within the flower, such as a closed carpel where ovules are protected throughout megasporogenesis, double fertilization and embryo formation, together with the development of the carpel into a fruit, provided a new means for seed protection, dispersal and colonization of new habitats (Pabón-Mora et al. 2014; Gonçalves 2021).

Cactaceae are a relatively recent flowering plant lineage that has evolved to cover a large range of environments, particularly arid and semi-arid ecosystems. This family has an estimated age of 35 million years (Arakaki et al. 2011), and recent studies suggest that it is still in process of diversification (Magallón et al. 2019). Cacti are one of the most representative groups of plants in the American arid and semi-arid ecosystems with fascinating morphological, ecological and physiological adaptations to hydric stress, such as reduced leaves, spines (modified leaves), a voluminous cortex to store water, stem succulence and a terminal flower seemingly sunken into vegetative tissue in what has been called a *flower-shoot* (Mauseth 2006). Diverse growth forms and pollination guilds across different species of Cactaceae may have provided a competitive advantage in terms of survival, reproduction, and the ability to expand their range (Hernández‐Hernández et al. 2014), resulting in the outstanding stem diversification observed in the family. In contrast the overall structure of its flowers has remained quite constant, with changes involving mainly variations in organ number, size and color (Britton and Rose 1920; Buxbaum 1953; Anderson 2001; Schlumpberger 2011; Almeida et al. 2013; Rosas-Reinhold et al. 2021).

Interestingly, while succulence is present in many species of the diverse families that comprise the order Caryophyllales (Cuénoud et al. 2002), such as the Portulacaceae, Basellaceae and Didiereaceae, the floral features in Cactaceae are at odds with those that characterize many of the other succulent families, namely, the presence of two involucral bracts in the flower, and five and rarely more perianth elements (Hofmann 1994; Vanvinckenroye and Smets 1996, 1999; Cuénoud et al. 2002). Additionally, cacti flowers develop a pericarpel and multiple petaloid elements with a spiral arrangement (Ronse de Craene 2022) that arise before the multistaminal androecium derived from a stamen ring primordium (Hofmann 1994; Leins and Erbar 1994). Ring meristems have arisen many times independently in different angiosperm orders such as the Ranunculales, Proteales, Fabales, Malvales, Caryophyllales, among others (Corner 1946; Endress 2006; Kong and Becker 2021).

The “pericarpel” describes the tissue enclosing the inferior ovary (Buxbaum 1953) in cacti flowers. The ontogenetic nature of the pericarpel has been motive of debate among scholars, as for some authors the pericarpel has a receptacular origin (Almeida et al. 2018), to others it is homologous to a hypanthium (Schlumpberger 2012), while yet others consider it axial tissue derived from the shoot (Boke 1964). Thus, although the term pericarpel has been amply used by cactologists, it lacks a consensus with regards to which structures originate and comprise it. This is likely in part because boundaries between the pericarpel and floral tissue -in particular the inferior ovary- are very diffuse and hard to piece apart. As a means to investigate the ontogeny of this tissue, the use of anatomy techniques and construction of developmental series (Sharawy and Khalifa 2018) aid in the understanding of its potential structural novelty (Muller and Wagner 1991) within Cactaceae (Caryophyllales) and angiosperms.

Since most cacti have an inferior ovary, most morphological research has been focused on understanding the nature of carpel formation and fruit formation post-fetilization (Tiagi 1955; Boke 1964, 1966, 1968, 1980; Ross 1982), not early pericarpel development. Cactaceae’s inferior ovary is the outcome of the floral meristem remaining convex during organogenesis and changing at the end of flower development (Soltis et al. 2003). Before the gynoecium primordium emerges, the periphery of the floral meristem is expanded and elongated producing a cup-shaped space in the center, giving way to epigynous flowers with an inferior ovary.

The pericarpel shares morphological and anatomical features with cacti shoots, such as the presence of photosynthetic and succulent tissue, while many species have leaves with axillary buds (areoles) with trichomes and spines on the external surface of this structure (Mauseth 2006, 2022), while other species can be nude with extrafloral nectaries (Mauseth et al. 2016). Furthermore, pericarpel can be pigmented or green with stomata. Anatomically, the pericarpel is composed of an epidermis, hypodermis, cortex and vascular tissue (Fuentes-Pérez et al. 2009), similar to the cacti shoot. These similarities between the pericarpel and vegetative shoots have been interpreted as evidence for the homologous nature of these structures, thus cacti flowers have been construed to be partially buried within a shoot, thus being called a *flower-shoot* (Mauseth 2016). The developmental processes taking place during reproductive unit formation in cacti have been scantly studied and the ontogenetic venues that give way to the *flower-shoot* are poorly understood. Information is also lacking regarding the identity of tissues that seemingly derive from axial tissue (at least part of the pericarpel) and those derived from the floral meristem.

In this contribution we present a developmental series that spans from the emergence of the shoot apical meristem (SAM) to the transition to a floral meristem (FM), the initial differentiation of the flower primordium and the establishment of all the organ types present in the flowers of *Disocactus speciosus* (Cav.) Barthlott and *D. eichlamii* (Weing.) Britton & Rose, two species with contrasting morphologies observed principally in the color of tepals, number of tepals, stamens and carpels, as well as the size and shape of the pericarpel. We discuss our findings in the context of floral developmental evolution in Caryophyllales and point to anatomical cues that could help understand the ontogenetic origin and posterior development of the pericarpel and scrutinize the *flower-shoot* concept. In addition, we compare developmental patterns in both species allowing us to understand the origin of the morphological differences between them.

## Material and methods

### Sample collection and anatomy

**Fig. 1:**
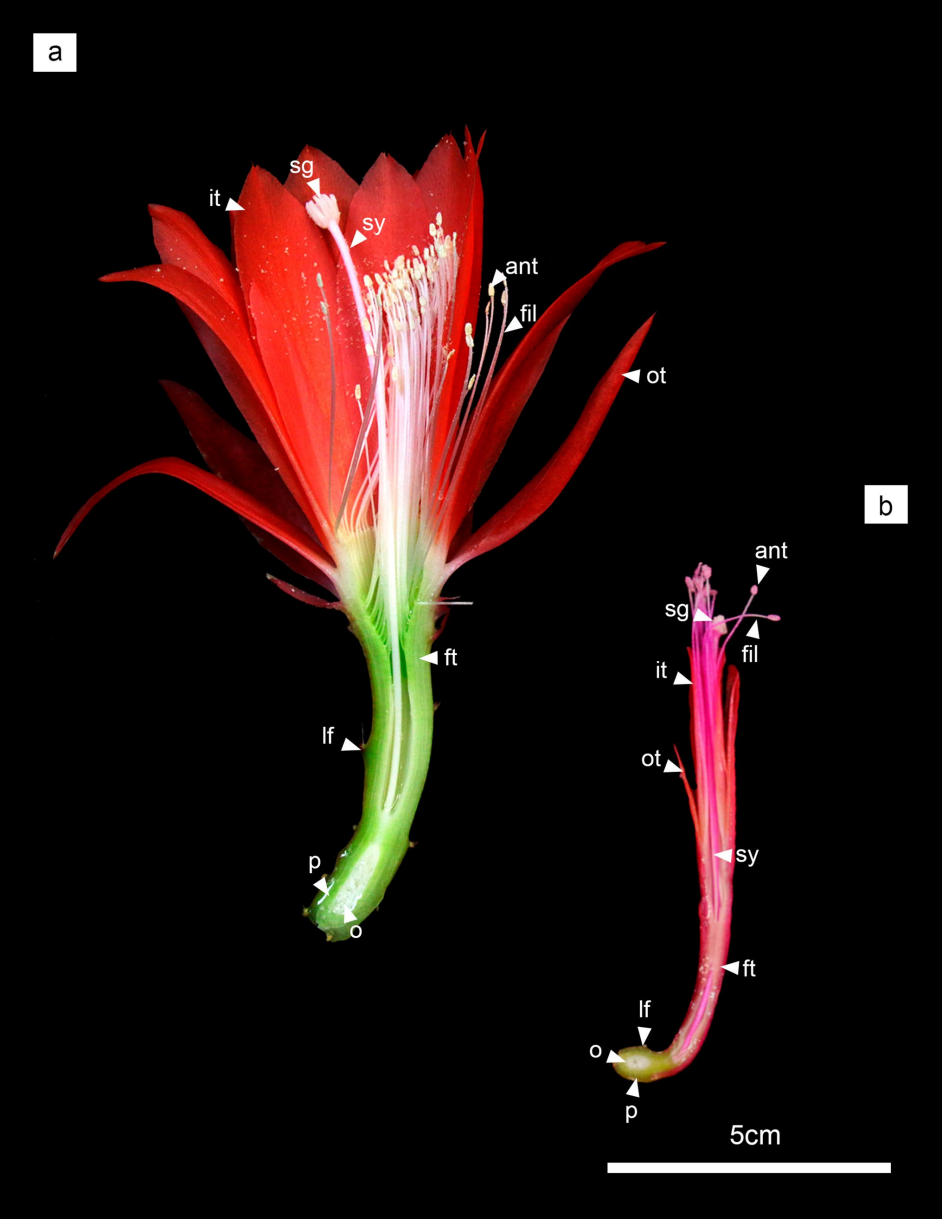
Flowers in anthesis showing several differences in floral morphology. **a** *D. speciosus*. Flowers in this species have a long tube and inferior ovary, funnel-shaped and a multistaminal androecium (∼200). The perigonium is composed by a spiral series gradation of sepal-tepaloids (outer tepals) to red petal-tepaloids (inner tepals), positionally close to leaves that give the appearance to be part of the perigonium. **b** *D. eichlamii*. Flowers in this species are tubular with a median size of 5 cm and during anthesis the tepals are not extended. The flower is composed of 12 tepals and no more than 20 stamens, in this flower leaves are reduced and located in the pericapel. ant = anther, fil = filament, ft = floral tube, it = inner tepals, lf = leaf, o = ovary, ot = outer tepals, p = pericarpel, sg = stigma, sy = style

*Disocactus speciosu*s and *D. eichlamii* belong to the Hylocereeae tribe, a group characterized by plants with hemi epiphytic and epiphytic life forms. *Disocactus* is a monophyletic group that occupies a derived position in the Hylocereeae (Cruz et al. 2016) with a distribution range from Mexico to Central America. One of the most interesting features in *Disocactus* is its flower diversity, which includes broad variation in flower color, shape, size and organ number. The two species selected here are closely related but show contrasting morphologies (Fig. 1) and geographic distribution. *D. speciosus* grows along Mexico and Central America while *D. eichlamii* shows a restricted distribution in Guatemala. *Disocactus speciosus* and *D. eichlamii* samples were collected from the proximity of the Reserva del Pedregal de San Ángel (REPSA) at Universidad Nacional Autónoma de México (UNAM), in Mexico City and from the epiphytic cacti collection of the Botanical Garden, IBUNAM, respectively. *D. speciosus* and *D. eichlamii* develop numerous flowers (~ 100 each plant) between January to March making these two species a good system to study flower development. A total of 15 flower buds ranging from 1 mm to 1.5 cm in size (at 1 mm intervals) were collected, as well as 15 areoles where meristematic activity was evident, as suggested by changes in color pigmentation of trichomes, but without any flower bud emerging. Samples belong to 5 different plants in *D. speciosus* and 1 plant in the case of *D. eichlamii*. Both flower buds and areoles were collected in triplicate in both species for each size category. Samples of both species were immediately fixed in FAA (ethanol, distilled water, formaldehyde and acetic acid in a ratio of 50:35:10:5 respectively) and put in vacuum for 60 min. The FAA solution was removed after 48 h in the agitation table. The materials were washed with distilled water.

The fixed areoles and flower buds were dehydrated in a tert-butyl alcohol series (35–100%, absolute, Sandoval Zapotitla et al., 2005), embedded in paraplast (Leica) using a paraplast infiltrator (Leica, HistoCore Arcadia H-C) at 60 °C and serially longitudinally sectioned at thickness of 7 μm using a rotary microtome (American Optical 820). Subsequently, mounted slides were stained with safranin-fast green (Johansen 1940), and examined under the light microscope, in average we analyzed 10 longitudinal sections per stage before establishing the 9 most relevant stages presented in these ontogenetic series. Slides were photographed under a bright-field microscope (Zeiss Axioscope) equipped with a digital camera (PANASONIC imxCMOS), using the Rising View of AmScope software. Contrast and light of microphotographs were edited in Photoshop CS6. Quantitative data like areole length, width of shoot apical meristem and floral meristem, ovary wall and length of floral bud were calculated using ImageJ (1.53 version).

### Scanning electron microscopy

Samples for the scanning electron microscope (SEM) were collected and fixed as per the method described above. After tissue fixation, flower buds at different developmental stages were dehydrated in an ethanol series (10–100%), critical point-dried with CO_2_ (EMITECH K850), mounted on aluminum stubs, and sputter coated with gold (QUORUM Q15OR). Images were taken using a scanning electron microscope (Hitachi SU1510, 10 KV) with a digital camera attachment. Contrast, light and coloring of scan electron microscopy micrographs were edited in Photoshop CS6.

## Results

### Ontogeny of the floral axis in *Disocactus speciosus* and *Disocactus eichlamii*

**Fig. 2:**
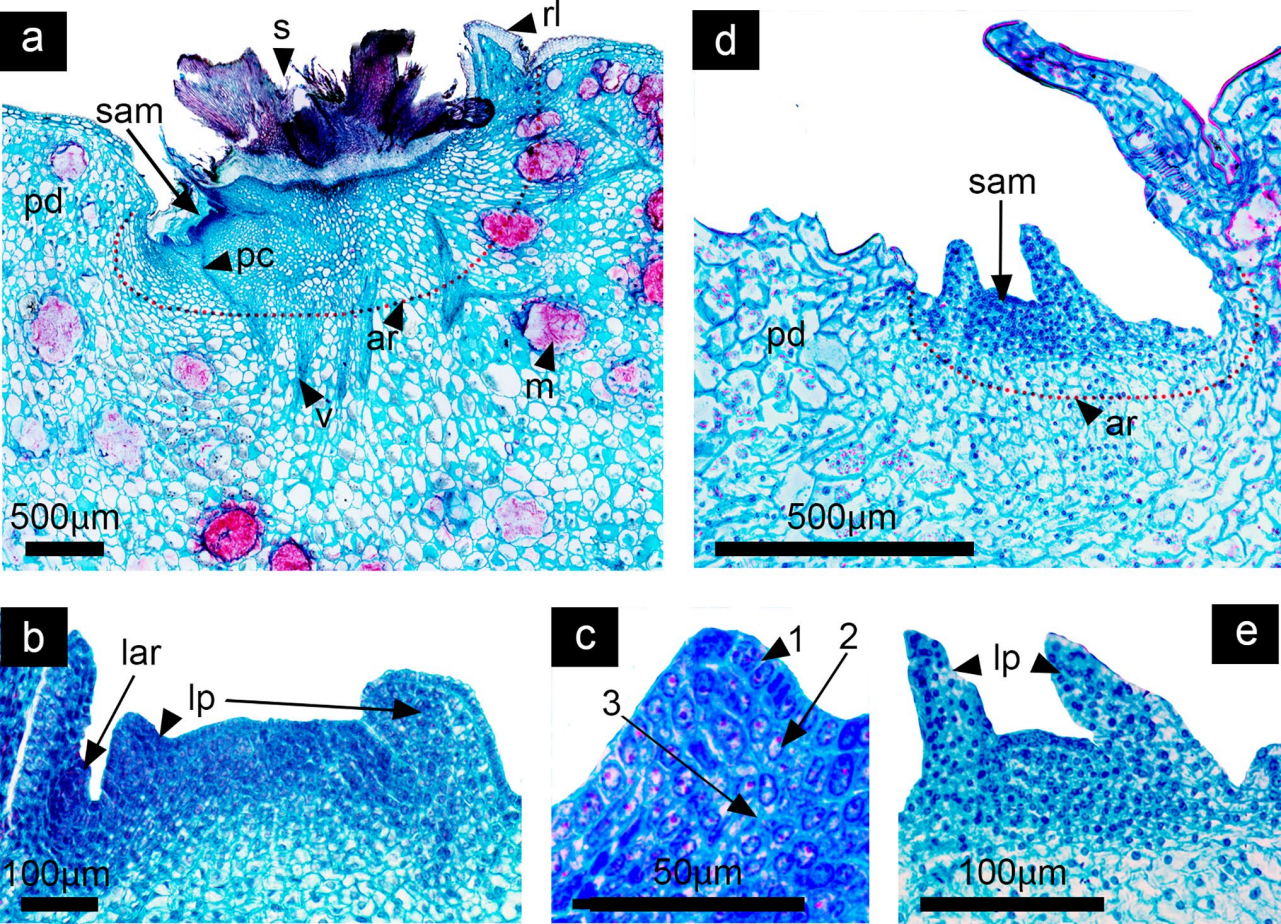
Stage 1: Activation of a meristem within an areole. *D. speciosus* (a, b and c); *D. eichlamii* (d, e). **a** Longitudinal section of the median region of the areole (ar) immersed in the podarie (pd) where it is possible to observe the spine (s) and the activation of a meristem that generates a flattened SAM. **b** Magnification of the SAM, where leaf primordia differentiate (lp). An areole primordium in the axil of a leaf is shown with a black arrow (lar). **c** Magnification of a leaf primordium in the meristematic dome stage where particular cellular strata (labeled 1, 2 and 3) can be observed. These cells are small and bear big nuclei with dense cytoplasm. From this strata, different tissues of the leaves develop. **d** Longitudinal section of the mid region. From the surface of a small areole (ar) a flattened SAM differentiates, flanked by leaves. **e** Close up to the SAM, where leaf primordia (lp) are visible. Note the different size between the SAM in *D. speciosus* b) and the SAM in *D. eichlamii* e). m = mucilage cells stained in red, pc = procambium, pd = podarie,, rl = reminiscent caulinar leaf, v = vascular bundle, lp = leaf primordia

**Fig. 3:**
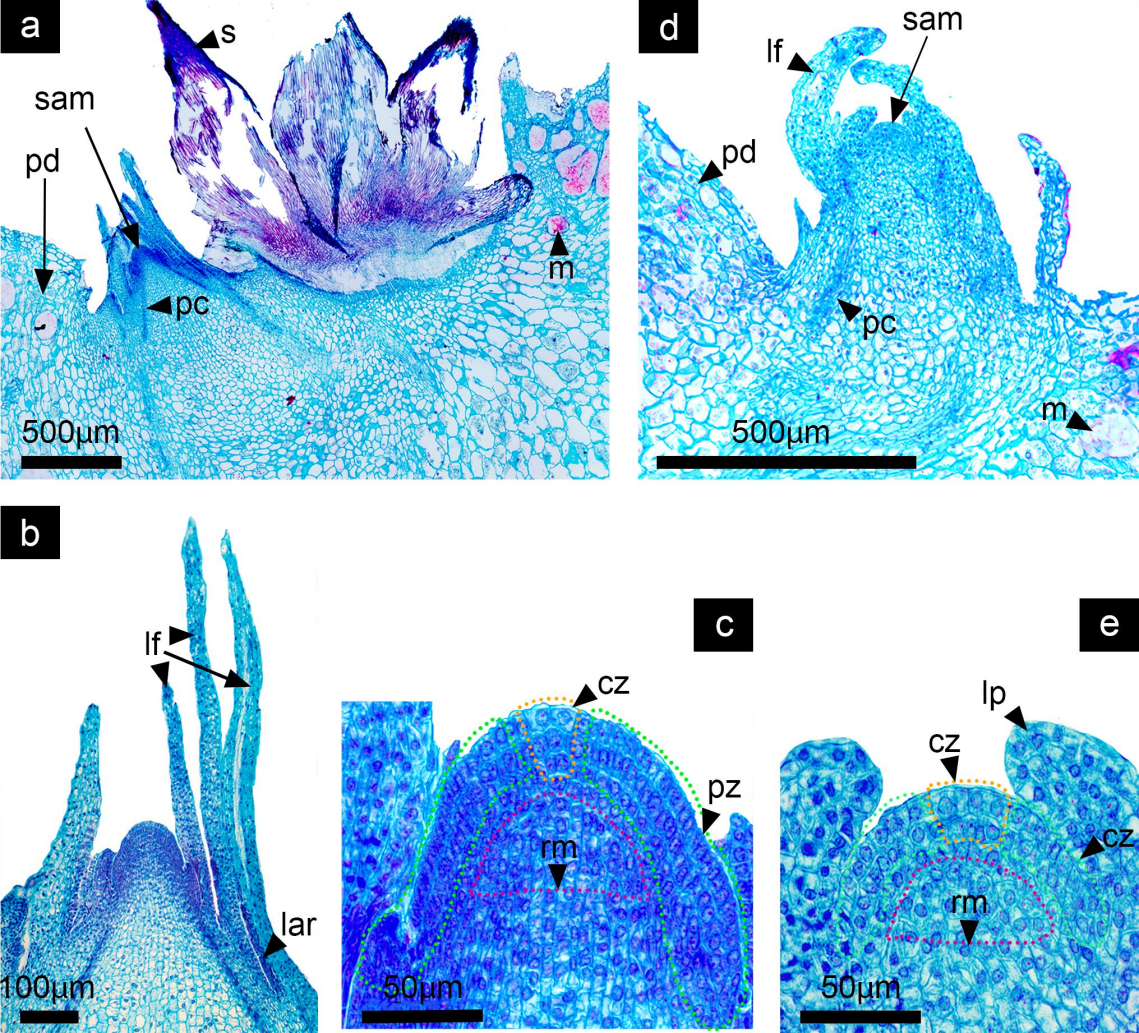
Stage 2: Elongation and differentiation of leaves enclosing the SAM. *Disocactus speciosus* (**a**,** b**,** c**) and *D. eichlamii* (**d**, **e**). **a** Longitudinal section of the podarium median region and areole. leaves (lf) grow until they cover the SAM. **b** Magnification of SAM and surrounding leaves. The SAM acquires its characteristic dome shape; note that older (external) leaves bear areole primordia (lar) at the axile. **c** Magnification of the SAM were different cell strata can be visualized; the central zone (cz, dotted yellow line), the peripheral zone (pz, dotted green line) and the ribbed meristem (rm, dotted pink line) are well defined. **d** Longitudinal section of the mid region of an areole. The SAM grows upwards, protruding above the podarie (pd) and is covered by developing leaves (lf). **e** The SAM acquires a domed shape which is divided into the central zone (cz) from where cells migrate to the other zones; the peripheral zone (pz) from where leaf primordia (lp) differentiate and the rib meristem (rm) below, from where ground tissue will form. m = mucilage cells, pc = procambium, s = spine, v = vascular bundles

**Fig. 4:**
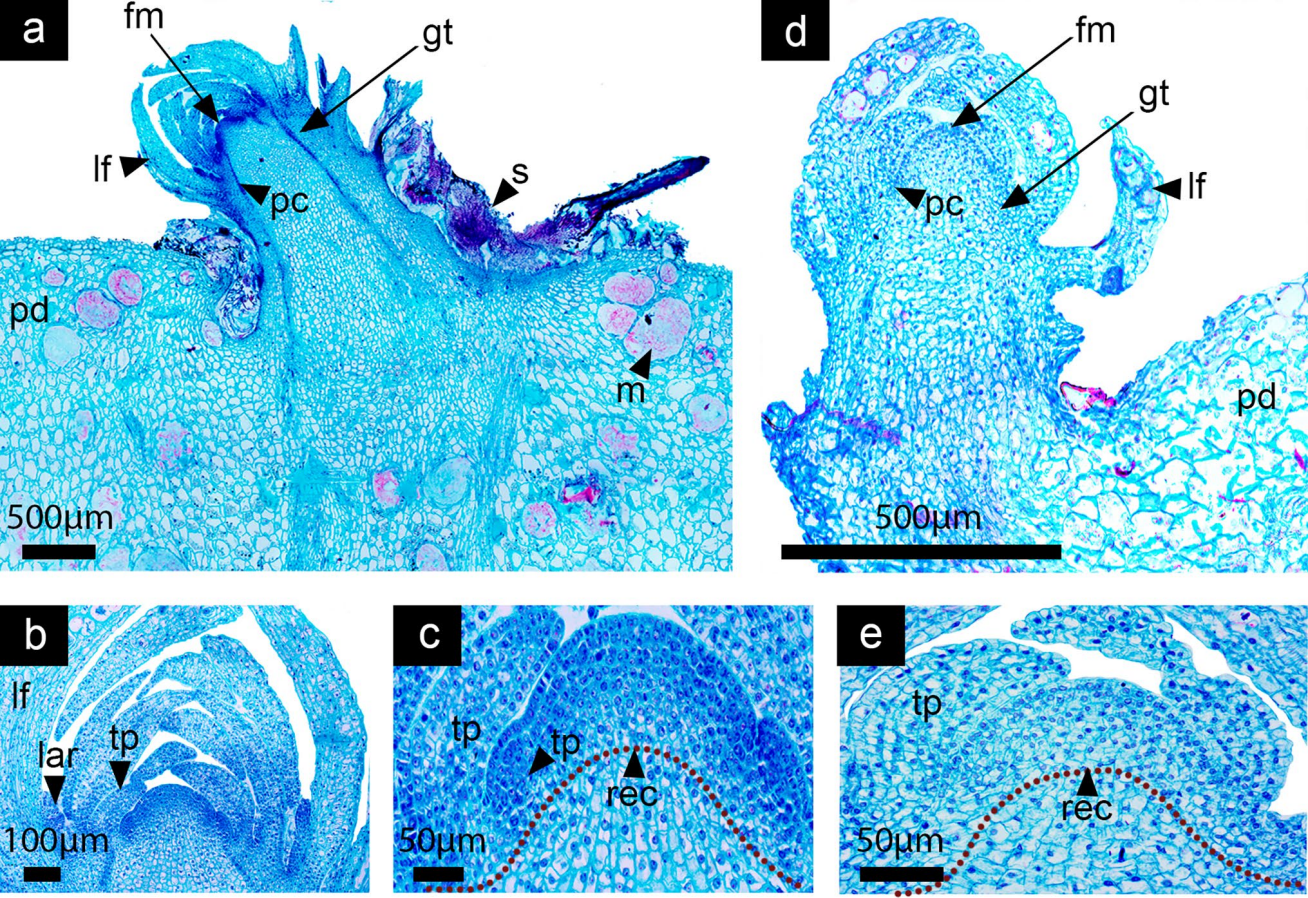
Stage 3: Differentiation of tepal primordia. *Disocactus speciosus* (**a**, **b**,** c**) and *D. eichlamii* (**d**,** e**). **a** Longitudinal section of median section of floral axis. **b** Magnification of the expanding FM; the emergence of tepals is marked with leaves (lf) enclosing the floral meristem. **c** Magnification of tepal primordia (tp) adjacent to the FM. Cell divisions in the strata 1 and 2 are similar to those in the laminar structures (leaves) differentiated previously. **d** Longitudinal section of the median region of the reproductive axis. A dome-shaped FM with well-developed leaves (lf) that enclose it can be observed. Inwards, tepals (tp) are present. **e** Magnification of the FM where the central zone and meristematic cells are present in its center, thus the transition to a FM has taken place. fm = floral meristem, gt = ground tissue, m = mucilage cells, pc = procambium, pd = podarium, rec = receptacle, s = spines

**Fig. 5:**
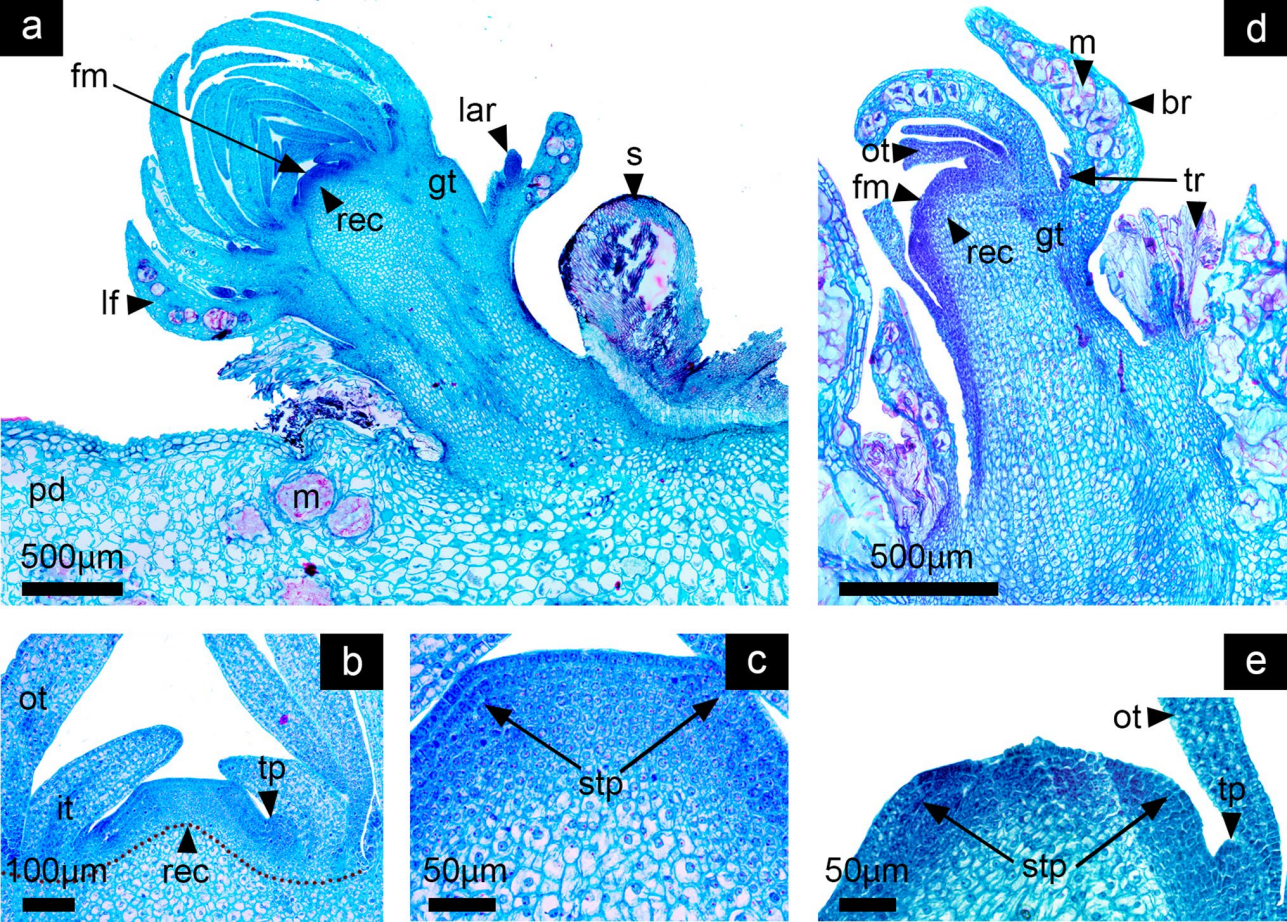
Stage 4: Flattening and further differentiation of the FM. *Disocactus speciosus* (**a**,** b**,** c**) and *D. eichlamii* (**d**,** e**). **a** Longitudinal section of the median region of the floral axis. Leaves (lf) with associated areoles and trichomes (lar) are evident in the outermost of the *flower-shoot*, while tepals are devoid of such structures. **b** Magnification of the floral meristem which is flattened. The inner tepals (it) and outer tepals (ot) are apparent, as well as a tepal primordium (tp). Below, the receptacular tissue (rec) starts to expand. **c** Close-up of the FM, marking the region where stamen primordia (stp) will develop from the ring meristem. **d** Longitudinal section of the median region of the reproductive axis. A flattened FM can be observed (fm), with differentiating tepals visible on its periphery. These tepals are morphologically distinct from the leaves (lf). The outermost leaves have a tuft of trichomes on their axils (tr). Big mucilage cells-stained red are apparent along the leaves. **e** Magnification of the FM. Note that on the angle formed by the flattened FM primordia that will form stamens is starting to differentiate. gt = ground tissue, m = mucilage cells, pd = podarium, s = spines

**Fig. 6:**
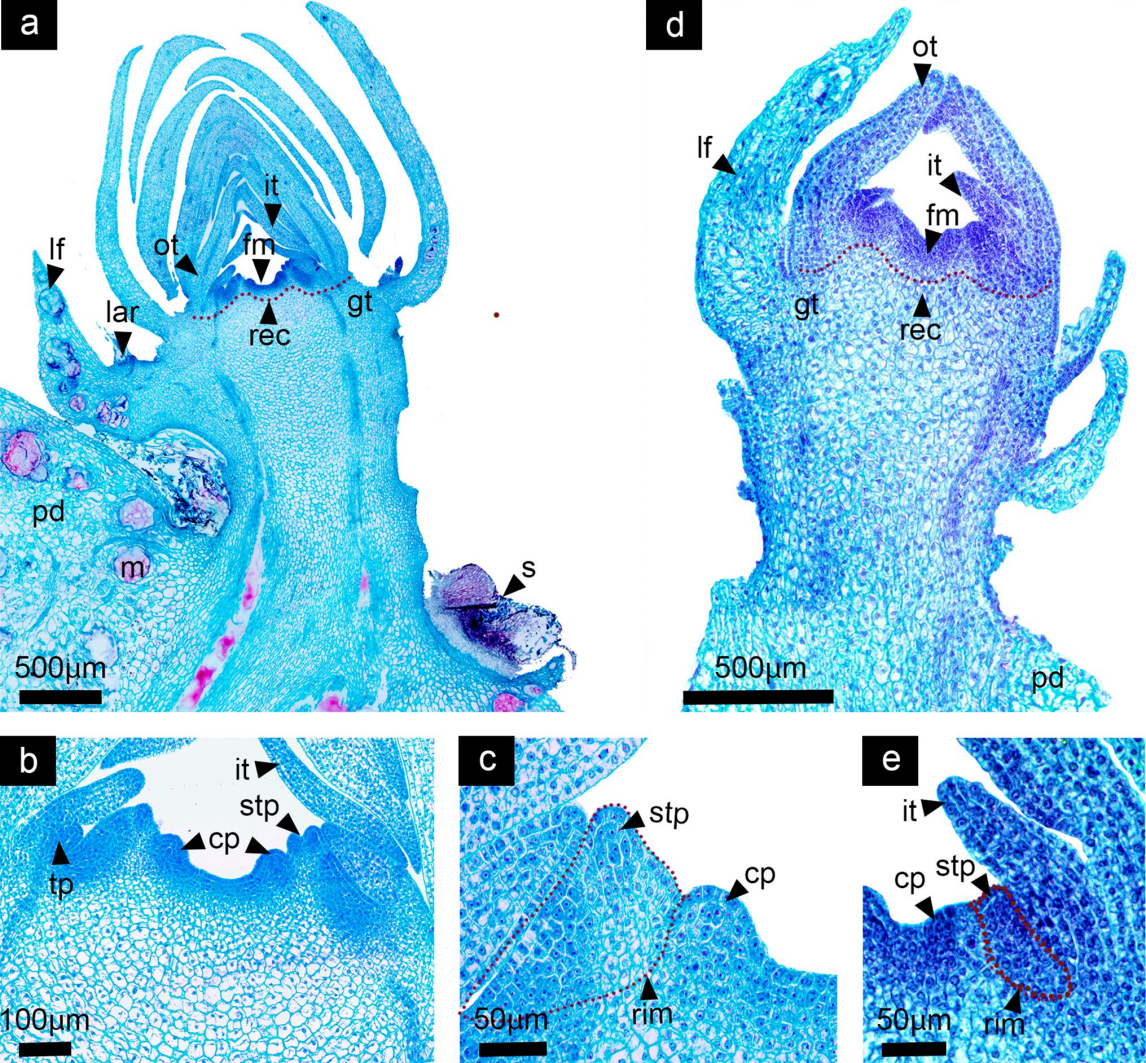
Stage 5: Staminal ring meristem differentiation and carpel development. *Disocactus speciosus* (**a**, **b**,** c**) and *D. eichlamii* (**d**, **e**). **a** Longitudinal section of the median region of the floral axis, where the formation of a staminal ring meristem (rim) and the differential growth of the tissue that surrounds the carpels primordia are apparent. (gt) **b** Magnification of the floral meristem where the carpel primordia (cp), staminal ring meristem and stamen primordia (stp), as well as internal tepal primordia (it) can be distinguished. **c** Magnification showing the limits between the ring meristem (rim, red dotted line) from where stamen primordia differentiate (stp) and the carpels (cp). **d** Longitudinal section of the median region of the reproductive axis. From the outside inward, leaf (lf), outer tepal (ot), inner tepal (it), stamen (stp), carpel (cp) primordia and the ground tissue (gt) are visible. **e** Magnification of the insertion site of the floral organ primordia, where the ring meristem (rim) has been outlined with a red dotted line and the stamen and carpel primordia have been marked (stp, cp, respectively). fm = floral meristem, lar = areole of the leaf, m = mucilage cells, pd = podarium, rec = receptacle, s = spines

**Fig. 7:**
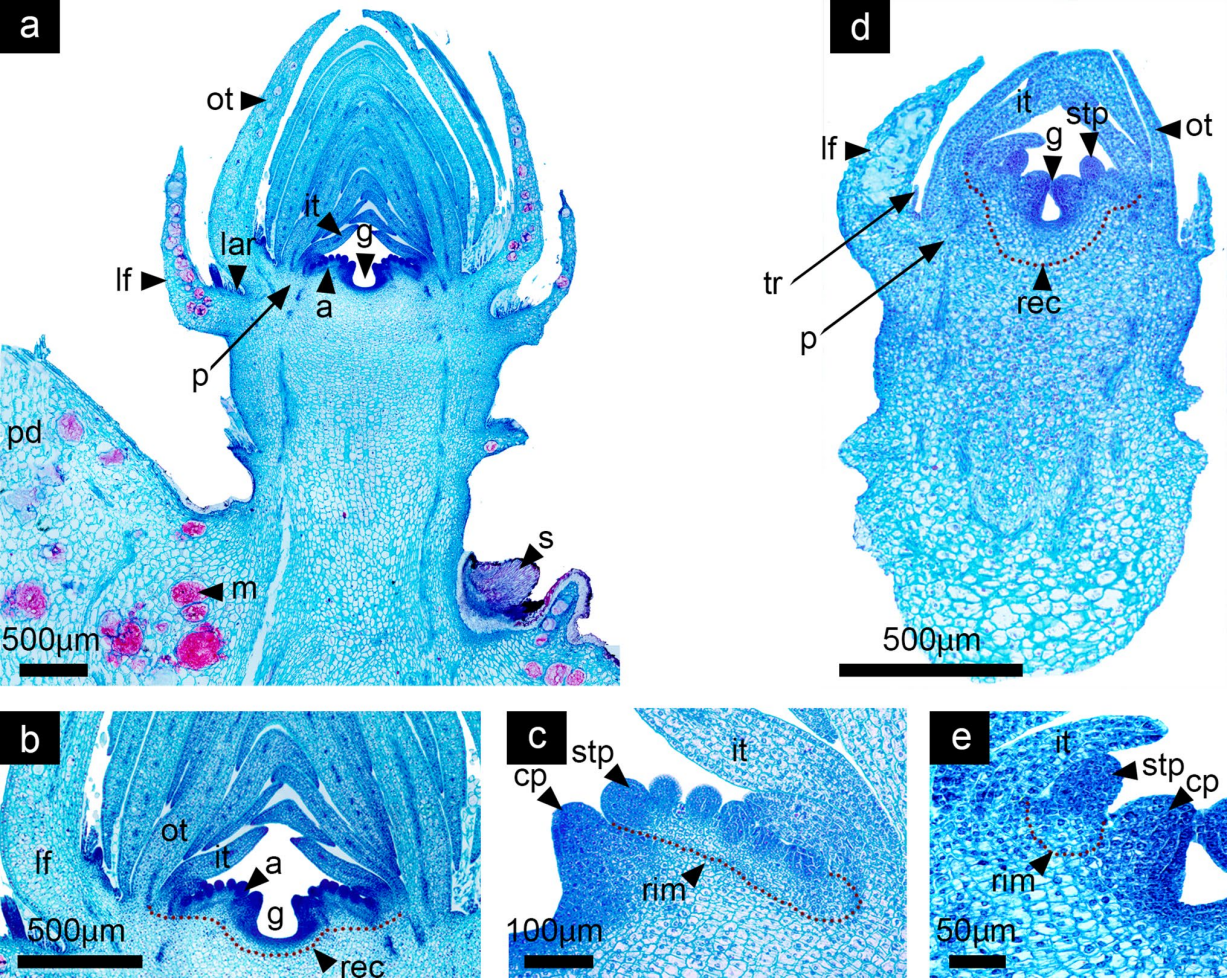
Stage 6: Ovary cavity formation and ring meristem expansion. *Disocactus speciosus* (**a**, **b**, **c**) and *D. eichlamii* (**d**, **e**). **a** Longitudinal section of the median region of the floral axis. At this moment it is possible to distinguish the pericarpel (p). The ring meristem comprising the androecium (a) is clearly distinguishable, as well as the gynoecium (g) **b)** Magnification of the floral meristem, where the receptacle is marked with a red dotted line.**c** Stamen primordia (stp) from the ring meristem (rim) are marked, as well as the carpel primordia (cp). **d** Longitudinal section of the median region of the reproductive axis. Floral organ primordia have continued elongating and a restricted number of stamen cycles (stp) is visible, as well as the pericarpel (p). **e** Magnification of the receptacular tissue (rec), which proliferates upwards through intercalary growth, pushing the stamens and tepals (it) up. Note the small ring meristem (rim). lar = areole of the leaf, m = mucilage cells, ot = outer tepals, pd = podarium, tr = trichomes

**Fig. 8:**
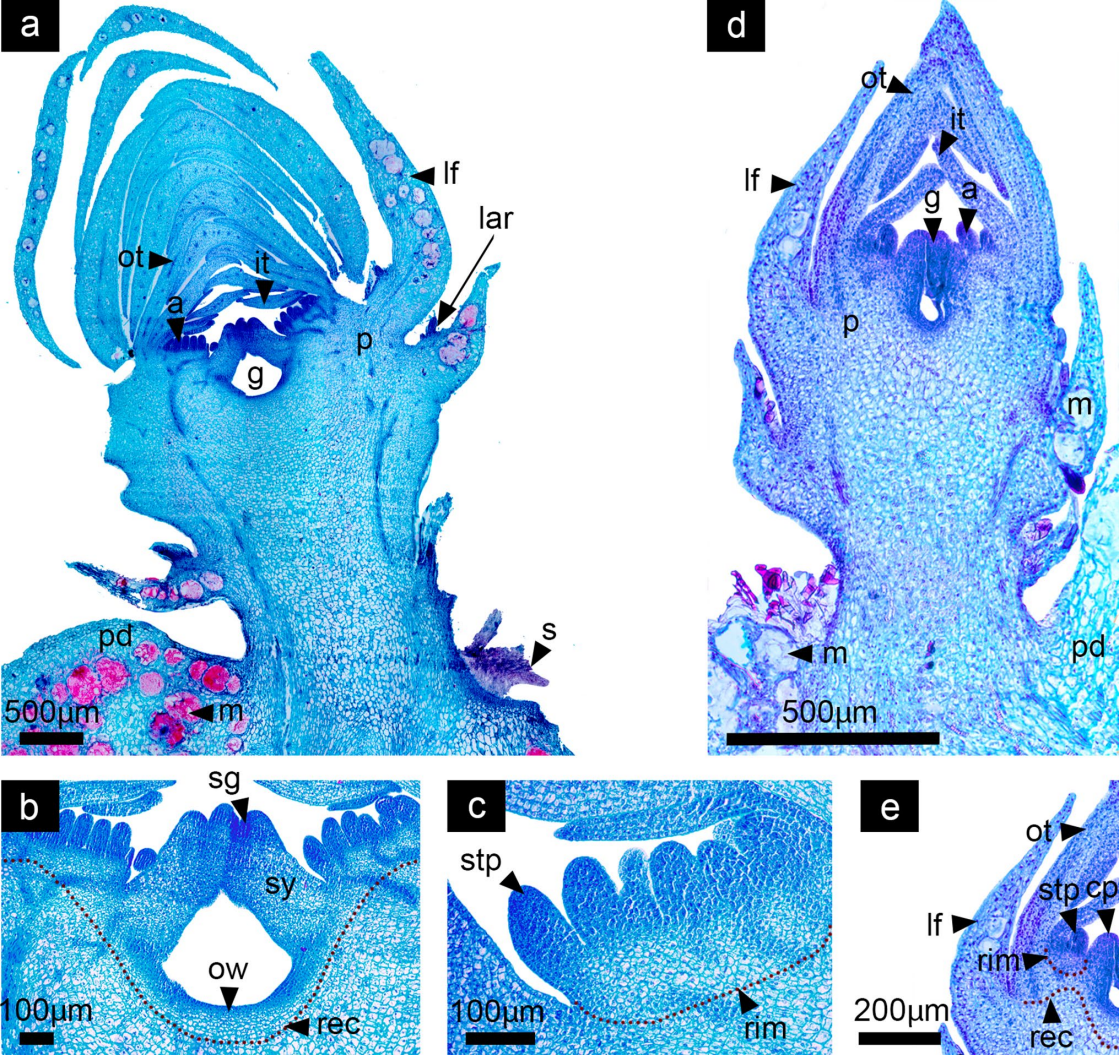
Stage 7: Expansion of pericarpel. *Disocactus speciosus* (**a**, **b**, **c**) and *D. eichlamii* (**d**, **e**). **a** Longitudinal section of the median part of the floral axis. The tepals enclose the floral apex (ot, it), while the pericarpel commences to surround the inferior ovary (g). **b** Magnification of the center of the floral primordium. The developing style (sy) and stigma (sg) are now apparent. The ovary wall (ow) is distinguishable from the underlying cells and the limits of the receptacle (rec). **c** Magnification of the massive staminal ring meristem (rim), where numerous stamen primordia develop (stp). **d** Longitudinal section of the median region of the reproductive axis. Tepals (ot, it) enclose the reproductive organs, the pericarpel (p) can be observed which surrounds the ovary in development.** e** Magnification of the small and restricted staminal ring meristem (rim) and receptacle (rec) where a stamen (stp) and a carpel primordium can be observed (cp). lar = areole of the leaf, m = mucilage cells, pd = podarium

**Fig. 9:**
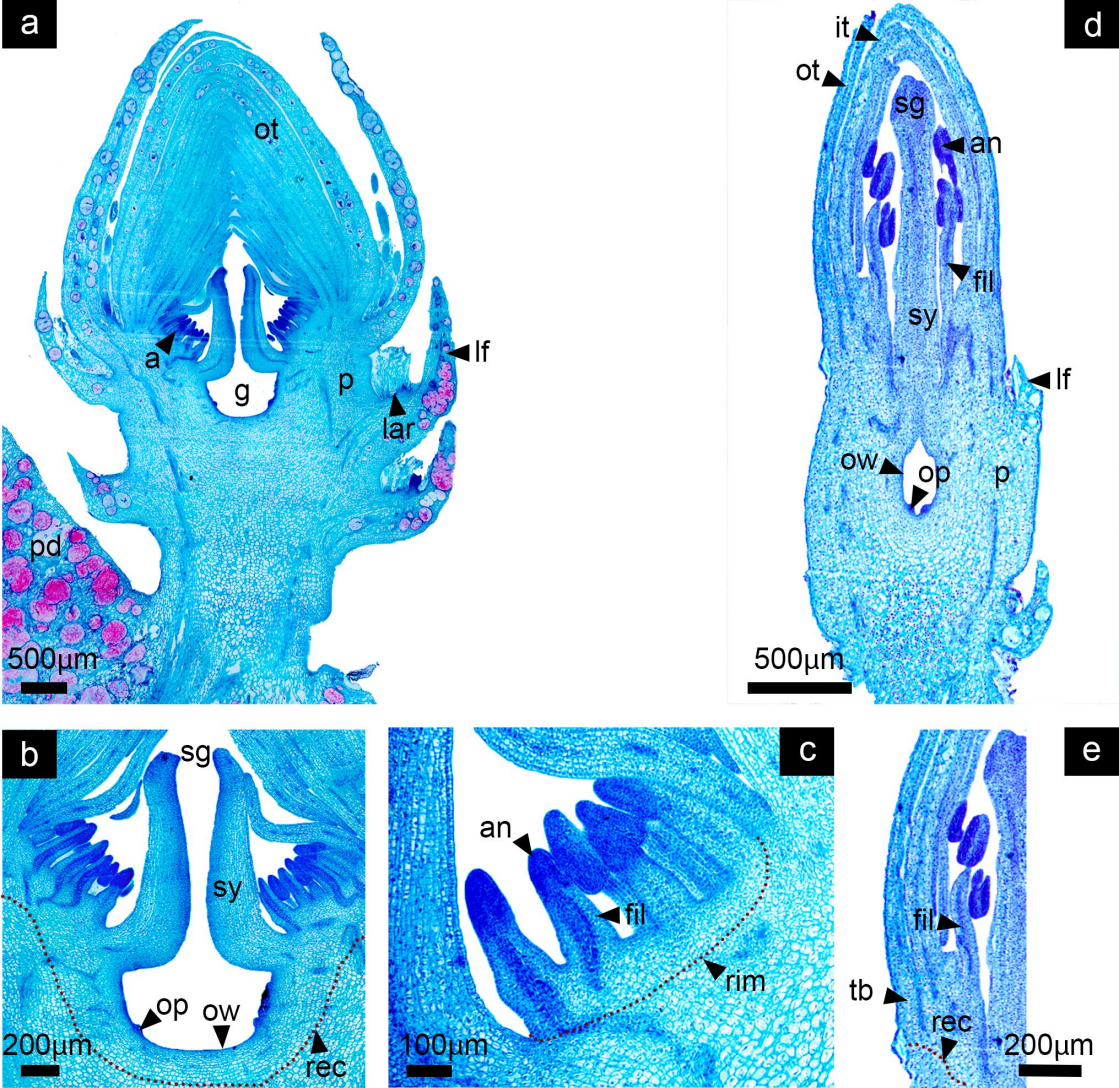
Stage 8: Floral organ growth, differentiation and elongation. *Disocactus speciosus* (**a**, **b**, **c**) and *D. eichlamii* (**d**, **e**). **a** Longitudinal section of the median region of the floral axis. In this section the continuum between the pericarpel (p) and the receptacle (rec) is evident; additionally, the floral organs are rapidly differentiating and elongating. **b** The ovary where the inner ovary wall (ow) and developing (op) ovules in a parietal position are apparent on the margins. **c** Magnification of a section of the androecium; a red dotted line indicates the ring meristem and underneath the growing receptacular tissue. Note that the filaments (fil) and anthers (an) are clearly differentiated in older stamens. **d** Longitudinal section of the median region of the reproductive axis. All floral organs have differentiated and elongated. From the inside outward, the gynoecium is composed of an unilocular inferior ovary. Stamens and filaments are now adnate at the tube. **e** Magnification of the receptacle, where the tepal bases (tb) are merged with the filaments of the stamens; this tissue will give rise to the floral tube. a = androecium, lf = leaf, lar = areole of the leaf, g = gynoecium, it = inner tepals, ot = outer tepals

**Fig. 10:**
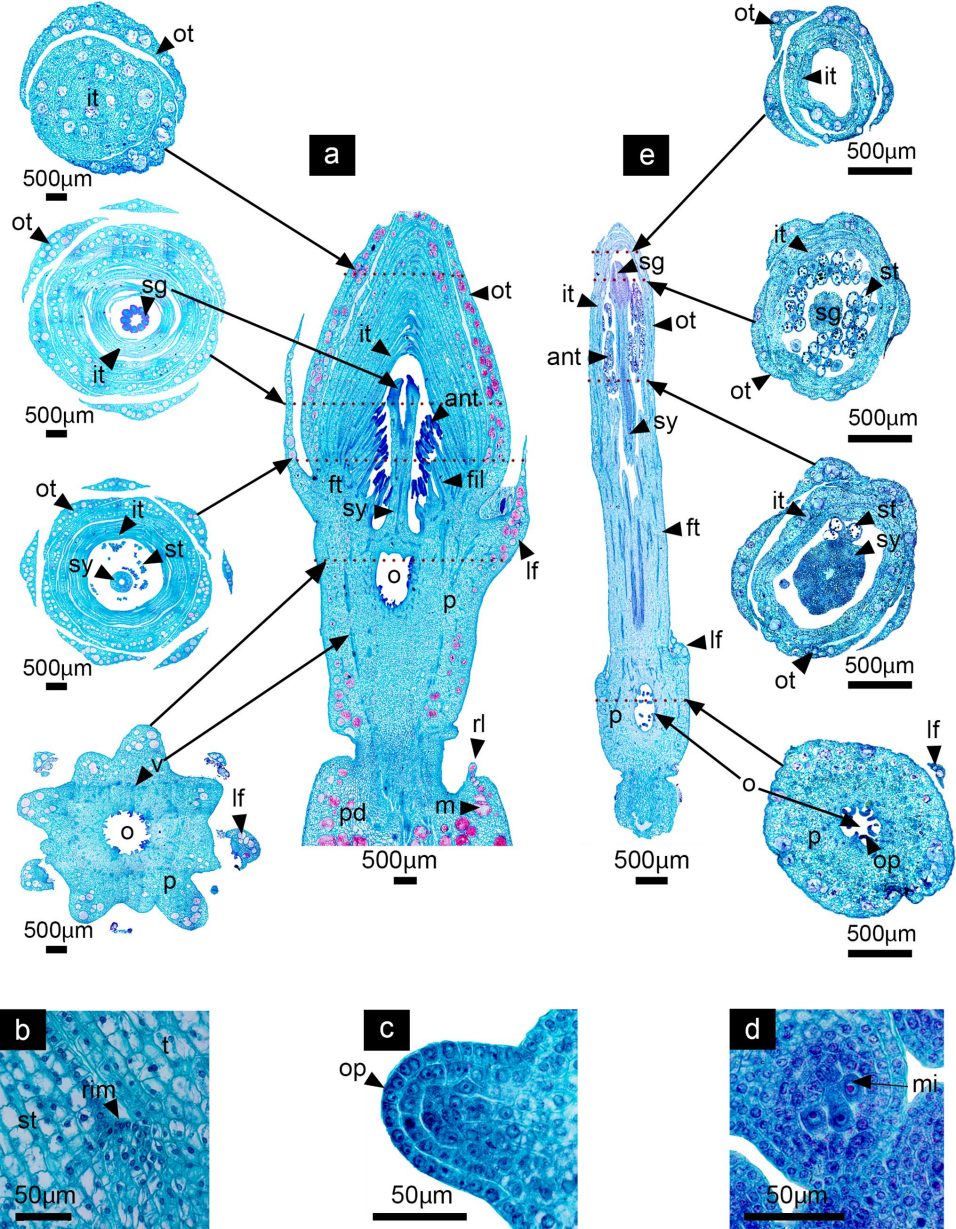
Stage 9: Elongation of the inner floral organs. *Disocactus speciosus* (**a**, **b**, **c**) and *D. eichlamii* (**e**). **a** Longitudinal section of a median region of the floral axis, together with cross sections in different parts of the floral axis (marked with red dotted lines). From the inside out, the ovary is completely formed (o), while the different carpels have fused together to enclose the syncarpous ovary and form the style (sy) and stigma (sg) composed of 9 lobules. The latter is still in the process of postgenital fusion. Developing ovules can be seen in parietal position (ov). Numerous stamens in different stages of maturation can be seen. Inner tepals (it) appear thinner than outer ones (ot). The continuum between the leaves (lf) that comprise the pericarpel (p) is apparent. This fusion is part of the pericarpel. Mucilage cells (m) stained in red are similar to those observed in the podarium (pd). **b** Magnification of the outer section of the ring meristem (rim) where meristematic tissue between a stamen (st) and an inner tepal (it) is still active. **c** Magnification of the ovary where ovule primordia (op) develop. **d** Transverse section of the microsporangium, where the microspore cells (mi) are observed. **e** Longitudinal section of the median region of the reproductive axis where the elongation of the floral tube (ft) and floral organ maturation is observed. From the outside inwards, and from the proximal to the distal region of the flower, the leaves (lf) remain restricted to the pericarpel section always associated to an areole, with a much smaller size than the tepals (it, ot), while no well-developed podaria are present. The pericarpel remains restricted to the ovary portion of the gynoecium, where the development of ovules (op) is taking place. A continuum of parenchyma from the inner epidermis of the ovary Toward the pericarpel is visible, as is the case in *D. speciosus*. The floral tube (ft) has elongated and seems to comprise the fusion of tepals and stamen filaments tissue (fil). In the distal portion of the tepals, these remain free. In the stamens, anthers are tetrasporangiate and have two thecae, where pollen is starting to mature. The semisolid style (st) with transmission tissue and the stigma (sg) is composed of 6 lobules

As in most Cactaceae species, *D. speciosus* and *D. eichlamii* develop solitary flowers differentiated from areoles located along the branches of the stem. Early flower development was analyzed and divided into four different phases that represent specific transitions during floral development. Stages 1 and 2 are included in the phase “A”, which begins with a period of vegetative growth and ends before the transition to a floral meristem. During this phase the areole -which is considered a short shoot with many meristems- becomes activated and the presence of a shoot apical meristem (SAM) can be observed (Fig. 2). The SAM can develop into a shoot or a reproductive floral meristem (undistinguishable at this stage). Also, it is possible to observe intense proliferative activity in peripheral zones, as well as in the meristematic rib. The developing SAM bears the typical features described for this structure, having small central cells with enlarged nuclei, the presence of a central zone with a lower meristematic activity, as well as the formation of leaves which grow in an acropetal manner in a continuous and accelerated growth around the SAM reaching a specific number in a spiral sequence (Fig. 3). This Phase ends with the transition of the SAM to a floral meristem (FM). Phase “B” includes stages 3 to 5, where the transition to a FM has occurred and the differentiation of the floral meristem into organ-specific primordia takes place, while the floral meristem becomes convex in the site where the ovary will develop. During this phase the floral axis differentiates in both species in a continuum composed by vegetative tissue derived from the SAM and floral tissue (tepals, stamens and carpels) derived from the FM. The tissue where the floral organs are inserted corresponds to a receptacle. The vegetative tissue from the SAM that forms a continuum with the receptacle with not clear limits. This vegetative tissue will grow differentially enclosing the ovary in later stages of development forming the composite structure, which will be called the pericarpel. We consider that at this phase, given that there is not a fully developed ovary, the underlying vegetative tissue cannot be called pericarpel, thus we consider the use of the term ground tissue to describe the structure under formation in this moment of development (Figs. 4, 5, 6). As part of the process of floral meristem differentiation, a conspicuous staminal ring meristem is formed in *D. speciosus* from where numerous stamen primordia will differentiate in a centrifugal order. In contrast, in *D. eichlamii* a small ring meristem is observed, thus limitanting the number of stamens that will develop. In this phase, carpel primordia develop within the gynoecium in both species (Fig. 6). Phase “C” comprises stages 6 to 8 which are characterized by the initiation of organ-specific tissue differentiation. Until this phase we can observe a proper pericarpel, i.e., a tissue (originally derived from the SAM) that covers the ovary, which in turn is marked by the appearance of carpel primordia. Also, intercalary growth in the receptacular tissue (derived from the FM) produces the elevation of tepals and stamens, which gives the appearance of a tubular flower and produces the inferior ovary (Figs. 7, 8). The last Phase “D” includes stage 9, which represents the last part of the period of early flower development. In this phase, cellular elongation and differentiation are evident in all floral organs, while the pericarpel grows. Additionally, the differentiation of other structures takes place such as the floral tube, the pericarpel, the nectary chamber and the development of ovules and pollen. Further intercalary growth allows for the elongation of the pericarpel and floral tube (Fig. 9). One of the major differences between the two species analyzed here is the ontogenetic origins of the floral tube as in *D. eichlamii* we observed the formation of a floral tube (Fig. 10b, 12g,h,l)*,* through the fusion of tepal bases and the adnation with the stamen filaments giving way to a stamen-petal tube, while in *D. speciosus* the floral tube is a receptacle tube composed externally by vegetative tissue bearing leaves and areoles, and internally covered by floral tissue derived from the adnation of stamen filaments (Figs. 1 and 10).

### Phase A. Initiation of vegetative growth of the SAM

#### Stage 1: Differentiation of the SAM and formation of leaves

In *Disocactus speciosus* and *D. eichlamii* as in other epiphytic cacti, the branches are composed of podaries and ribs called phylocladodia. The podarium is mainly composed of ground and photosynthetic parenchyma, with an abundance of mucilage cells (Fig. 2a, d), The areole is localized on the tip of the podarium and is 2 mm wide in *D. speciosus* (Fig. 2a, red dotted line) but in *D. eichlamii* the areole (ar) is highly reduced, and approximately 500 µm wide (Fig. 2d). The areole meristem gives way to trichomes and spines that cover it. Associated with the areole is a small, reduced leaf. In this first stage, the areole is activated, and the development of a SAM starts. This SAM is slightly flattened, with a width of 135 µm in *D. speciosus*, and of 93 µm in *D. eichlamii.* Leaf primordia differentiate from the SAM on a spiral pattern (Figs. 2b, e). These primordia begin like a protrusion in the second layer of cells in the SAM (L2) produced by periclinal divisions (Fig. 2c). In this stage for *D. speciosus* it is possible to observe the presence of small domes in the leaf axils, composed by a set of undifferentiated cells (Fig. 2c). These domes are similar to axillary buds that in Cactaceae differentiate into new areoles that will go on to produce spines and, which will have the potential to develop organs such as flowers, shoots and even roots. In contrast, in the axils of leaf primordia of *D. eichlamii* no axillary buds were observed; these could have been reduced to the point of disappearing (Fig. 2e).

#### Stage 2: Elongation of leaves enclosing the SAM

In both species, the SAM’s “meristematic dome” continues and is enclosed by spirally arranged leaves (Fig. 3). This meristem resembles a typical shoot apical meristem in other angiosperms. In the central area of the SAM that encompasses the cell layers corresponding to the central zone (cz), peripheral zone (pz) and ribbed meristem (rm), a group of cells of larger size can be distinguished (Fig. 3c, e). The latter zone maintains the totipotentiality in this meristem during this period. In *D. speciosus* in the axils of leaves in development it is possible to observe the formation of areoles while in *D. eichlamii* these are not apparent. In this stage the SAM protrudes over the podarie (Fig. 3a, d). The leaves which are covering the SAM start to elongate and differentiate as well as the areole primordia in the leaves axiles (Fig. 3b). While in *D. speciosus* leaves are elongated with visible areole primordia (Fig. 3b), in *D. eichlamii* leaves are shorter with no visible areoles (Fig. 3d). At this moment it is also possible to observe the procambium which later will give rise to the vascular tissue.

### Phase B. Transition to a FM, differentiation of floral organ primordia and early receptacle formation

#### Stage 3: Transition to a FM and differentiation of floral organ primordia

Both species at this stage of development transition from a SAM to a floral meristem (FM). Leaves (approximately three per spiral) that have reached a bigger size, showing mucilage (polysaccharides) cells and a heterogeneous mesophyll can be observed (Fig. 4a, d). The differentiation of floral organ primordia begins, as marked by the emergence of tepals from the outer region of the FM that partially enclose the meristem apex (Fig. 4b, d). Tepals development follows the same spiral sequence as the leaves. In *D. speciosus* the FM dome is wider than the SAM, reaching 230 µm, while in *D. eichlamii* it is 160 µm (Fig. 5c, e). In this stage the totipotential zone with active cell divisions begins to be restricted.

#### Stage 4: FM flattening and further differentiation of floral organ primordia

On this stage in both species the FM is flattened, and the differentiation of organ-specific primordia is more evident (Fig. 5a, d). The axis of the FM is increasingly expanded by the differential activity of cell division. The outer leaves form a spiral series with the tepals (Fig. 5b, e). Both types of structures can be differentiated by the size of the mesophyll, the stage of development and in the case of the leaves, by the presence of areoles in their axils which develop spines and clusters of trichomes in *D. speciosus* (Fig. 5a), while in *D. eichlamii* leaves bear trichomes but not spines (Fig. 5d). Toward the periphery of the FM, the outer tepals grow and the mesophyll, protoderm and procambium tissues elongate. During this stage, and after the initiation of the tepals, the FM becomes a wide, low disk with vague angles (Fig. 5a, d) and the first stamen primordia arise from a ring meristem doing so in centrifugal order, without any apparent relation with the elements of the perigonium (Fig. 5c). Below the flattened meristem, several layers of small cells form an incipient receptacle (Figs. 5b, e).

#### Stage 5: Staminal ring meristem differentiation and carpel development

In this stage every cell of the FM has acquired a specific identity (Fig. 6a, d). While tepals still develop in the outermost region of the FM, an incipient staminal ring meristem is now apparent, preceding the carpel primordia appearing, at the same time the FM started to become convex (Fig. 6c, e; 12c, i) product of cells on the margins which are under rapid division and growth, giving an appearance of a cavity in its center. Within the carpel, the innermost cell strata will give rise to the internal ovary wall (Fig. 6b). The insertion site of floral organs is known as the receptacle. Below, ground tissue derived from vegetative tissue starts to grow differentially, surrounding the carpels (Fig. 6a, d). This developmental pattern is similar in both species.

### Phase C. Enclosement of the ovary by the pericarpel

#### Stage 6: Ovary cavity formation and ring meristem expansion

In both species, the reproductive axis has expanded in size and width, while a clear distinction between vegetative tissue composed by leaves inserted in the stem axis and a floral region with developing floral organs is visible (Fig. 7a, d). By now, even when early flower development had been very similar in both species, noticeable differences become more obvious. The perianth in *D. speciosus* is composed by 30–40 larger outer tepals and smaller inner tepals that enclose the reproductive organs (Fig. 7a), while the perianth in *D. eichlamii* is composed by 9–12 inner and outer tepals (Fig. 7d). Also, differences of the overall size of the flower primordium are evident (see scale bar in Fig. 7a, d). In both species the ovary is clearly inferior, the receptacle -where the tepals and stamen primordia are inserted- continues growing differentially in conjunction with the stem tissue by cellular divisions, elevating stamens and tepals above the carpels (Fig. 7b). In this stage the androecial ring expands and continues developing the stamens in centrifugal and asymmetric manner. Although both species show an androecial meristematic ring there are differences that distinguish them. In *D. speciosus* it is a massive ring, fully segmented where individual stamen primordia differentiate in a centrifugal manner; this ring meristem will remain active during later stages of development producing near to ~ 200 stamens (Fig. 7c). In contrast, in *D. eichlamii* the ring meristem is closed and smaller, restricting the number of stamens that will develop, thus only producing around ~ 20 stamen primordia (Fig. 7e). In both species it can be observed that the syncarpous ovary cavity forms through the congenital fusion of carpel primordia (Fig. 7a, d).

#### Stage 7: Expansion of pericarpel

At this stage, in both species, the pericarpel starts to surround the developing ovary. This portion of vegetative tissue internally forms a continuum with the receptacle and the inner ground tissue, while Toward the surface of the shoot, it forms a continuum with the foliar tissue of the leaves inserted in the stem (Fig. 8a, d). The leaves will be arranged differently depending on their relative position. In later developmental stages the apical leaves will be positioned above the pericarpel, partially enclosing the perigonium; the basal leaves will cover the pericarpel and will be located at its base (Fig. 8a) characterized by the presence of the areole. The tips of carpel primordia that form the ovary chamber give rise to the style and stigma. The ovary with a concave appearance enlarges due to intense proliferative activity in the ovary internal wall, in which cells with prominent nuclei are intensely stained (Fig. 8b, d). The androecium in both species starts to differentiate and elongate (Fig. 8c, d).

#### Stage 8: Floral organ growth and differentiation

During this stage of flower development in both species, the reproductive organs are in the process of organ-specific differentiation and growth. In *D. speciosus*, the multiple stamens grow while anthers and filaments are clearly distinct. In *D. eichlamii,* stamens have also differentiated into filament and anthers, however in this species, stamens seem to be in two cycles, likely due to the reduced ring meristem (Fig. 9). The receptacular region grows together with the underlying pericarpel, favoring the elevation of the bases of the androecium and perianth allowing the formation of the floral tube. The inferior ovary is further expanded, and the ovule primordia are visible, displaying a parietal placentation. The inner ovary wall is composed of epidermis and the outer wall is composed of a series of rows of quadrangular parenchymal cells. It is not clear if an outer epidermis exists, as the tissue seems to form a continuum with the ground tissue of the pericarpel (vegetative). In the distal region of the flower the style rises with the stigma in the tip (Fig. 9b). In *D. speciosus*, new stamen primordia are still differentiating from the ring meristem which continues active (Fig. 9c). In both species leaves continue to develop in the periphery of the pericarpel and floral axis, being smaller than tepals (Figs. 9a, d).

### Phase D: floral organ maturation and development of nectaries

#### Stage 9. Elongation of the floral axis

**Fig. 11:**
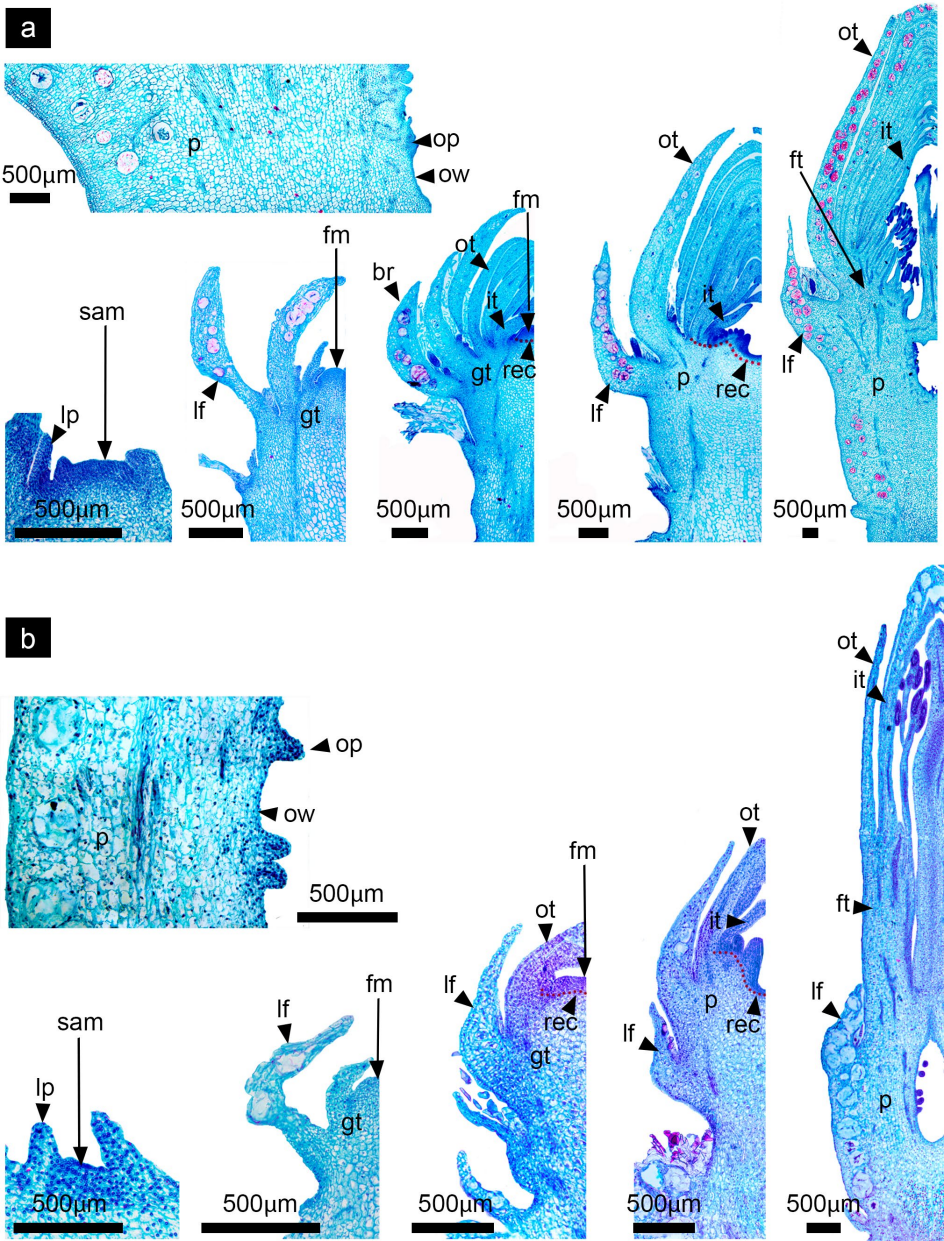
Comparison of pericarpel development in **a** *D. speciosus* and **b** *D. eichlamii*. In both species the tissue that will form the pericarpel derives from the SAM, which produces a series of leaves (lf) whose bases, inserted in the axis, will fuse to form the external vegetative section of the pericarpel. During the first stages, there is no well-developed ovary, thus, the vegetative tissue (ground tissue) that in those stages is still under the FM organ primordia later on forms the pericarpel. This developmental pattern occurs in both species as can be observed above. Once the ovary is developed, it becomes difficult to delimitate the pericarpel (p) tissue from the ovary wall (ow), therefore there is a continuum between floral-derived tissue (the ovary and receptacle) and vegetative tissue (the pericarpel). The main difference between the two species is that in *D. speciosus* the pericarpel continues to grow and forms the outer part of the floral tube (ft, Fig. 1), while in *D. eichlamii* the pericarpel is covering only the region where the ovary is located, and the floral tube is derived solely from floral tissue. it = inner tepals, ot = outer tepals, fm = floral meristem, op = ovule primordium rec = receptacle, sam = shoot apical meristem

In this last stage of development, in both species the floral axis has reached a length of approximately 1 cm all floral organs are well defined. At this moment, the pericarpel in both species is delimited internally by the collateral vascular bundles (Fig. 10a, e). The outermost part of this tissue is composed of the foliar tissue, which comes from the bases of the leaves which are inserted in the stem axis. The external ovary wall forms a continuum with the parenchyma tissue of the stem, while the inner wall is composed by a simple epidermis where ovules will differentiate. The style, the stigma and the ovary compose the gynoecium of both species. The style in *D. speciosus* is semisolid with transmission tissue and will eventually grow until it reaches 10 cm in length with a 9–10 lobed stigma. In *D. eichlamii*, the style is also semisolid, but shorter (4 cm in length), with a 5–6 lobed stigma. The stamens of *D. speciosus* derived from the ring meristem grow in a spiral and centrifugal manner. At this stage in this species the ring meristem is still active and new stamen primordia differentiate between the outer stamens and the inner tepals (Fig. 10b). In contrast, in *D. eichlamii* at this stage no new stamens are produced. In both species, the inner and outer tepals enclose the sexual organs, which also have a spiral disposition (Fig. 10a, e). In *D. eichlamii*, the bases of tepals are postgenitally fused, producing a floral tube, in contrast to the floral tube in *D. speciosus*, which derives from the growth of vegetative tissue and receptacle, bearing externally leaves, areoles and spines (Fig. 1). A common feature in both species is that the tissue lining the inside of the floral tube seems to derive from the adnation of stamen filaments to the tube (Figs.11c, 16b). In both species we observed the formation of the ovule primordia (Figs. 11c, d).

## Discussion

The study of flower development in cacti is a poorly explored field. The lack of developmental series for cacti species representative of the different lineages into which this family has been divided, has translated into uncertainties pertaining the ontogenetic origin of the different tissues that compose the so-called *flower-shoot* in this group. In this work we performed a developmental series that covered early development and organ-specific differentiation in two epiphytic cacti species with contrasting flower morphology: *D. speciosus* and *D. eichlamii*. Our series suggest that while these two species develop very similarly at the onset of the differentiation of the floral axis, both bearing terminal, sessile, single flowers, in later stages of development they diverge in both qualitative (the limits of the pericarpel and surrounding leaves) and quantitative ways (the number of series of tepals, stamens, carpels and ovules). These differences could explain the contrasting flower shape and final size in anthetic flowers, as well as the size of the resulting fruit.

In both species analyzed here, flower development is initiated with a period of vegetative growth, through the appearance of a SAM which produces lateral leaf primordia (Fig. 2); this stage has been considered by some authors as the moment of areole activation (Mauseth 2017) that precedes the formation of a reproductive axis. The nature of laminar structures observed in the *flower-shoot* has been the motive of debate; authors like Buxbaum (1953) describe them as bracts or scales. For Mauseth (Mauseth 2006, 2016, 2022), the laminar structures associated with the flower in Cactaceae are true leaves, as they develop from leaf primordia and have large lamina and abscission zones, developing areoles, which are homologous to axillary buds. Our observations in *D. speciosus* and *D. eichlamii* are congruent with Mauseth’s interpretations, where the formation of areoles at the base of the leaves during early stages of development, can be traced back to meristematic cells derived from the SAM (Fig. 11a). This developmental pattern observed in *Disocactus* is consistent with axillary bud development observed in *Arabidopsis*, where axillary buds come from the SAM (Grbic and Bleecker 2000). In *D. speciosus* it is possible to observe early on during development the differentiation of areoles producing trichomes and spines, however, during this same stage in *D. eichlamii* we could not observe the presence of spine primordia at the very reduced areoles in the axils of the leaves, only trichomes (Fig. 11b). This could be due to the overall compaction and reduction observed in this species with respect to *D. speciosus.*

In Cactaceae, while leaves are obvious in Pereskioideae, Maihuenioideae and Opuntioideae, in Cactoideae (Mauseth 2022) leaves are reduced to structures a few millimeters in size, in shoots hidden by the areoles (Mauseth 2022), but foliage leaves are still present in many flowers in the pericarpel and tube portion (Mauseth 2016). Our morphoanatomy and ontogenetic observations allow us to posit that flower-associated laminar structures in *D. speciosus* and *D. eichlamii* are true leaves, arranged spirally along the pericarpel axis until the transition to a floral meristem and the frontier with the perigonium (Figs. 10 and 11).

In leaves inserted in the flower basis in the last stages of development documented here, we could observe the differentiation of a parenchyma with elongated cells, similar to a photosynthetic palisade parenchyma which was more evident in *D. speciosus* (Fig. 10a, upper left). The palisade parenchyma is a common feature in cacti shoots, except for *Pereskia* s.l. and *Maihuenia* (Boke 1980; Mauseth 2006). This tissue is restricted to specific regions of the shoot denominated podarium, which are considered homologous to a modified leaf base (Boke 1980; Mauseth 2006). These thickenings at the base of leaves were observed in the *D. speciosus* flowers and to a lesser extent in *D. eichlamii* (Fig. 11a, b). We also observed that the base of the leaves inserted in the pericarpel become decurrent in later stages of development, thus, the exterior of the pericarpel is foliar in origin, while its interior is ground (parenchyma) tissue with isodiametric cells from the stem. This is particularly obvious in the last phase described here (Fig. 11a, last panel).

These observations from the tissue composing the pericarpel, are consistent with those made by other authors in the pericarpel of *Opuntia* (Fuentes-Pérez et al. 2009), where palisade tissue, collenchyma and stomata were reported; similar features were also documented in *Epiphyllum,* where an uniseriate epidermis, paracytic stomata, a chlorophyll cortex, crystals and secretory cavities were described (Almeida and Sartori-Paoli 2010). Furthermore, this suite of anatomical features has been considered evidence of the homology between the pericarpel and shoots. These traits are congruent with observations made in shoots of other species from the Hylocereeae tribe, where the *Disocactus* genus belongs (Martínez-Quezada et al. 2020). Thus, the traits described in the pericarpel of *D. speciosus* and *D. eichlamii* suggest that the ontogenetic origin of this structure can be traced back to the vegetative tissue originated from the SAM (ground tissue), derived in turn from an activated areole, although it is considered a pericarpel s.s. until the (inferior) ovary has developed (Figs. 9, 11). Furthermore, the developmental series presented here favor the hypothesis that the flower is not sunken from the onset into the underlying (and surrounding) vegetative tissue, but instead is covered by this tissue because of the differential growth rate between cells from the ground tissue (later the pericarpel) and the developing floral organs, including the ovary. Thus, while in mature flowers these can appear sunk into a branch (Mauseth 2006; Rosas-Reinhold et al. 2021) they do not start out like this.

**Fig. 12:**
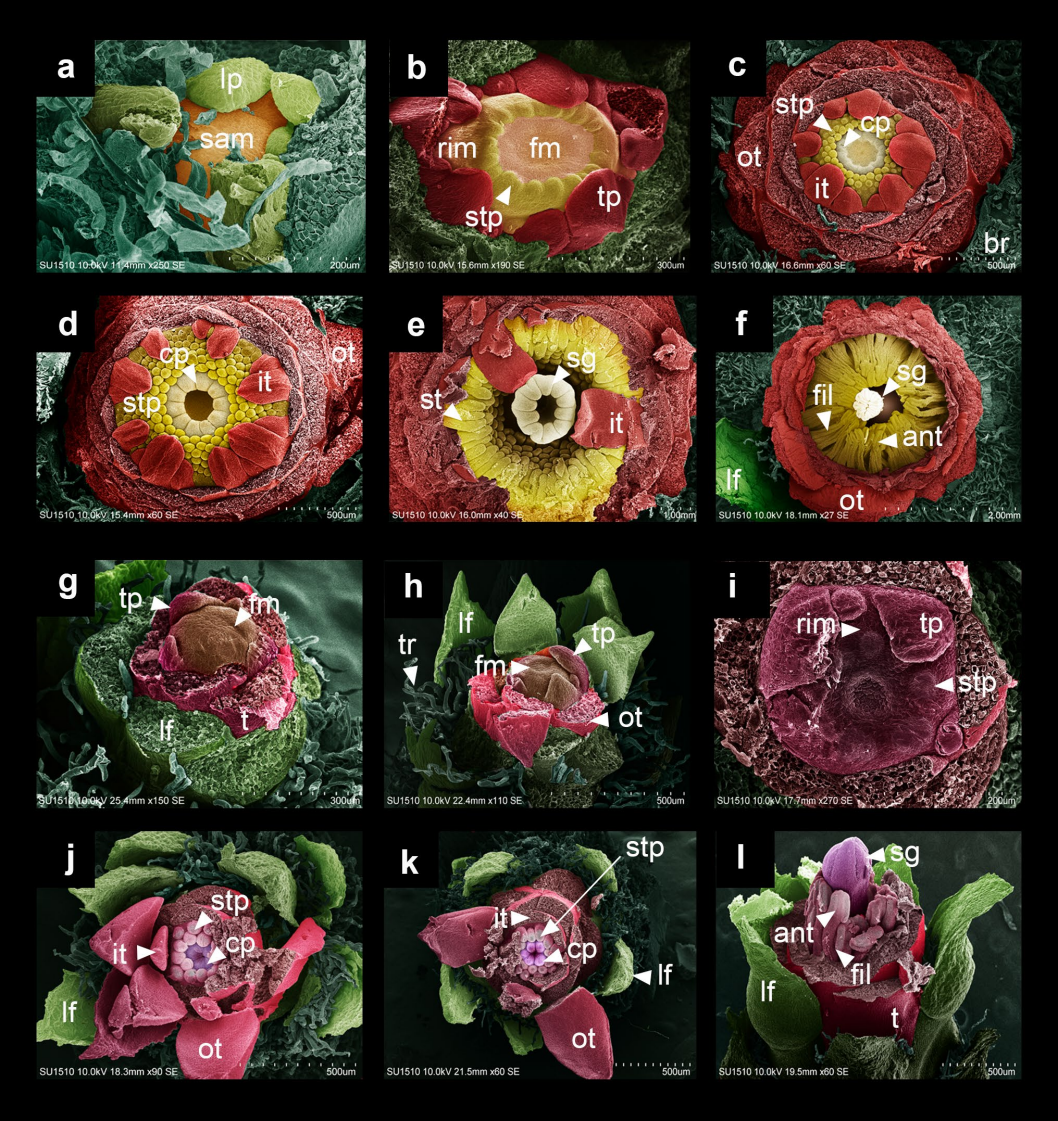
Scan electron microscopy photos of Different stages of flower development in *Disocactus speciosus* (**a**–**f**) and *D. eichlamii* (**g**–**l**) in polar view. **a** the SAM starts to flatten and is surrounded by leaves primordia (lp, colorized in green). **b** The FM has started to flatten, and the gynoecium primordia is apparent (cp), as well as a ring meristem (rim) with stamen primordia (stp) developing centrifugally. The center of the FM starts to become convex. Tepals are also evident (tp). **c** Several whorls of tepals are visible, as well as a well-developed fragmented ring meristem which produces stamens in centrifugal order. The carpels start to develop fused congenitally forming a syncarpous ovary. **d** In this stage the flower has started to elongate and a cavity between the stamen (st) whorl and the inferior carpels is evident, as well as the formation of the stigma (sg). The receptacle and the ovary walls are starting to the elongate (cp) and elevate, surrounding the gynoecium. **e** At this stage the stamen thecae and filaments are visible, while the stigma lobes continue developing (sg). Note the elongated thecae (st). **f** The stigma has been formed (sg) and the stigma lobes are well formed, while stamens bear well differentiated filaments (ant, fil). Note the various layers of tepals (ot, it partially removed) and leaves in spiral order, which are surrounded by trichomes that derive from the areoles associated to the base of each leaf (lf, green). **g** The floral meristem is observed with the presence of tepal primordia (tp) emerging from the periphery and the dome shape of the FM is preserved (fm). On the outside of the floral axis, leaves are visible (lf, colored in green) with trichomes (tr) in their axils. Tepals are already fused at the base. **h** The innermost part of the FM begins to flatten, and its center becomes slightly depressed (fm), like a donut shape. The areole leaves produce abundant trichomes. **i** Close up of the FM where the stamen primordia (stp) are present within a reduced and restricted ring meristem (rim), internal to the tepal primordia (tp). In the center of the developing flower, the carpels (cp) begin to form. **j** Spiral arrangement of the leaves (lf) and tepals (it, ot). Two cycles of stamen primordia (stp) are formed and inwards the carpel primordia (cp) will form 5 lobes of the stigma. **k** The floral organs grow and elongate. **l** The anthers (ant) and stigma (sg) develop rapidly and elongate. At this stage the tepals are completely fused at their base forming the outermost of the floral tube, which also begins to grow

Regarding the differences between the two species analyzed here, the most evident are the number of stamen and tepals, but also the number of leaves, carpels and ovules as well as overall flower size (Figs. 1, 12). These differences can be the result of floral organ series, which could be related with the starting size of the meristem (Bull-Hereñu et al. 2018) or to meristem maintenance. The molecular changes underlying the observed differences in merosity, could be related to species-specific changes in the expression of transcription factors homologous in function to *PERIANTHIA*, *ETTIN* and *SUPERMAN*, which act independently of the *CLAVATA/WUSCHEL* (meristem maintenance) pathway in the determination of floral organ number (Bowman et al. 1992; Running and Meyerowitz 1996; Sessions et al. 1997; Xu et al. 2018).

Polyandry in Cactaceae flowers (as in other Caryophyllales) has been suggested to be the outcome of a closed meristematic ring (Hofmann 1994; Ronse de Craene 2013). Ring meristems can be closed or fragmented, with a centrifugal or centripetal direction of stamen development (Kong and Becker 2021). In Cactaceae, ring meristems are reported as closed with a centrifugal development of stamens (Hofmann 1994; Leins and Erbar 1994), however only a small number of cacti species have been examined for this trait. *D. speciosus* shows a centrifugal stamen initiation, common in other cacti species (Ross 1982) but instead of bearing a closed ring meristem (Kong and Becker 2021), it appears to be a fragmented ring meristem, as the last stamens initiate after the carpels are advanced in their development (Fig. 12d–f), while tepal petaloids develop early and appear before the stamen ring primordium without obvious relation with the androecium (Fig. 12b). In cacti, the meristematic ring is closely connected with the gynoecium, as loss of carpels can lead to stamen loss, while an increase in carpel number is correlated with an increase in stamens (Ronse de Craene 2013). While meristematic rings have arisen multiple times in flowering plants, the genetic factors directing their development and maintenance are still unknown (Kong and Becker 2021). Both *Disocactus* species studied here possess a meristematic ring but *D. speciosus* will develop more than 200 stamens and 9–10 carpels, while *D. eichlamii* will bear 20 stamens and 5–6 carpels; in the latter, the small number of floral elements could be related with the size of the FM and the concomitant reduction of the ring meristem, which seems to restrict the number of stamens that will develop centrifugally and thus be a “closed ring meristem” (Fig. 12i–k). Androecial ring meristem with a centrifugal initiation is a feature commonly exhibited in Caryophyllales (Leins and Erbar 1994; Vanvinckenroye and Smets 1996, 1999; Brockington et al. 2013), like Anacampserotaceae and Portulacaceae which also are sisters to Cactaceae (Walker et al. 2018). Interestingly, these closely related families show different flower developmental patterns: they are pentamerous (only develop 5 petaloids), the ovary is superior and two involucral bracts cover the flower bud. In contrast, in Cactaceae we observe multiple elements in the perigonium and an inferior ovary enclosed by the pericarpel (vegetative tissue).

Further comparative studies of floral meristem size could explain whether the multiplication of floral organs is related with floral meristem size or with spatiotemporal changes in the expression of genes which have been related with organ multiplication, as well as variation in types of ring meristem in cacti species and in closely related families in Caryophyllales. It is interesting to note that the formation of a ring primordium instead of separate fascicle primordia, followed by a reduction in size of this ring wall until it disappears completely, have been proposed as the main steps in the lineages where simple androecia have evolved (Leins and Erbar 1994).

Another striking difference between the two species analyzed here is the patterning and ontogeny of the floral tube. In Cactaceae many flowers are tubular or funnel-shaped as a product of long floral tubes. These tubes sometimes are externally covered by leaves, spines and trichomes derived from areoles, hinting at their axial origin.

In *D. speciosus*, the floral tube is the product of intercalary growth of the outer vegetative tissue, while the internal portion of the tube is lined by tissue derived from stamen filaments which are adnate postgenitally to the tube (Fig. 10a). In contrast, *D. eichlamii* develops a stamen-sepal tube, being the outcome of the adnation of the bases of tepals and stamen filaments (Fig. 10b); the former comprising the outer part of the tube, while the latter line the internal portion. The presence of this type of floral tube in this species had been previously reported by Buxbaum (1953). Thus, while floral tubes are common in cacti flowers, these structures are not necessarily homologous and should be further studied because a greater diversity in the family can be observed.

A phenomenon that limits our ability to delimit the ontogenetic origin of the different tissues that compose a mature cactus flower, is the general lack of inflorescences, which if present, could allow us to trace the boundaries more easily between vegetative (SAM) or floral derived tissues. This seems to be a general restriction in Cactaceae, where most species have been described as baring either terminal or solitary flowers with some exceptions in *Pereskia* s.l (Leuenberger 1986) and *Myrtillocactus* (pers. obs.). Consistent with this observation, in both *D. speciosus* and *D. eichlamii* there is no evidence of a (transitional) inflorescence meristem, and the only morphological marker that allows us to differentiate the transition from a SAM to FM, is the initiation of tepal primordia. While the definition of *flower-shoot* implies a lack of clear boundaries between vegetative and floral derived tissues, in *D. eichlamii* we can clearly delimit these two types of tissues, making it an interesting case for further study. Despite the impending questions pertaining the ontogenetic pathways that enabled the formation of an inferior ovary enclosed in vegetative tissue -originating the pericarpel- this structures seem to be an important feature in the fruits of cacti, which develop a very unique and complex type of fruit called a *cactidium*, whose distinct feature is that its fleshy pulp is formed by the ovule funiculus that produces sugars which are enclosed in the foliar, non-maternally derived tissue of the pericarpel (Almeida et al. 2018). The peculiar fruit of Cactaceae, with no homologues in other angiosperm groups, has remained difficult to classify, leaving many open questions regarding the origin and development of the *cactidium*, an ongoing topic of debate among cactologists (Almeida et al. 2018). In this work we document the early development of the reproductive axis in two species of epiphytic cacti with contrasting flowers, discussing the origin of the pericarpel and its relationship with the portion of the flower that will eventually develop into a fruit. Future comparative studies of representative species with conspicuous or very reduced pericarpel (as is the case for *Opuntia* and *Mammillaria,* respectively) could shed light into the origin and evolution of this unique structure that has been instrumental for the dispersion of cacti in harsh environments, as well as for the colonization of new habitats.

## Conclusions

In both species evaluated here, the floral axis is composed by a continuum between a vegetative tissue (pericarpel) covering the flower organs. The external part of the pericarpel is composed from leaves bases inserted in the vegetative axis, thus, we propose that the outer part of the pericarpel is foliar. The inner portion of the floral axis could be vegetative and floral, but it is difficult to define morpho-anatomical limits between the ovary wall and the pericarpel. As the floral axis is very diverse across Cactaceae, we consider it necessary to evaluate other species in the different subfamilies in order to compare the development of this important structure across the family. Furthermore, analyzing early stages of development -such as the ones documented here- can help define which are common processes during floral axis ontogeny in cacti, and which are unique to particular lineages or species.

Lastly, our study shows how two closely related species can have qualitative and quantitative differences in flower development, which have implications in the final morphological features they display. Such as, flower size and organ number that can be related with the flower meristem size and the differences in the androecial ring meristem. Given that Cactaceae is a family with nearly 1,800 species, it is important to continue the analysis of flower and fruit development at early developmental stages as an additional means to fully understand the evolution of shape and floral organ identity in this charismatic group of angiosperms.

### Author contribution statement

Original idea: CGR-C and IR-R; Sample collection: CGR-C and IR-R; developmental histology: CGR-C; anatomy and microphotographs: CGR-C; scan electron microscopy photos: CGR-C and IR-R; data interpretation: CGR-C, IR-R, ES-Z and AP-N; wrote first draft paper: CGR-C, AP-N and IR-R; made a critical review of the draft paper: SA and ES-Z. All authors agree with the final version of the manuscript.

## Data Availability

All data generated or analyzed during this study are included in this published article.
